# Hot Douching in Sprains and Bruises

**Published:** 1835

**Authors:** 


### XIV.—Hot Douching in Sprains and Bruises.

It is not uncommon to see cold water applied forthwith to parts which have been strained or bruised, while a state of nervous irritation and reduced circulation, still exists in the general system. This practice we take it, is radically wrong. Some years since, a judicious non-medical acquaintance took some pains to impress upon us the results of his own experience in favor of hot bathing in cases of this kind. After having given the practice a "fair chance," we are enabled to speak of it in terms of decided approbation. It is especially adapted to persons of a nervous and irritable temperament. We have at this time a patient whose case is in point, and has, indeed, suggested the propriety of this notice. In jumping from a high fence, she lit upon the outside of her left foot, and when taken up was unable to stand. Considerable tumefaction immediately followed, with a total inability to move the ankle-joint. Extensive ecchymosis has since ensued, around and above and below the outer ankle. Being kept in a horizontal posture, with her foot over a tub, hot water was poured over it by the hour, and repeated as often as the pain recurred. She is now convalescent, without the slightest symptom of inflammation. We need scarcely add, that *should* inflammation ensue, in a case of this kind, the hot water would not be the best remedy, being, in reference to that pathological state, preventive not curative.

